# The role of multimodal cues in second language comprehension

**DOI:** 10.1038/s41598-023-47643-2

**Published:** 2023-11-27

**Authors:** Ye Zhang, Rong Ding, Diego Frassinelli, Jyrki Tuomainen, Sebastian Klavinskis-Whiting, Gabriella Vigliocco

**Affiliations:** 1 Experimental Psychology, University College London, London, UK; 2 Language and Computation in Neural Systems, Max Planck Institute for Psycholinguistics, Nijmegen, The Netherlands; 3 Department of Linguistics, University of Konstanz, Konstanz, Germany; 4 Speech, Hearing and Phonetic Sciences, University College London, London, UK; 5 Christ Church College, Oxford, UK

**Keywords:** Cognitive neuroscience, Human behaviour

## Abstract

In face-to-face communication, multimodal cues such as prosody, gestures, and mouth movements can play a crucial role in language processing. While several studies have addressed how these cues contribute to native (L1) language processing, their impact on non-native (L2) comprehension is largely unknown. Comprehension of naturalistic language by L2 comprehenders may be supported by the presence of (at least some) multimodal cues, as these provide correlated and convergent information that may aid linguistic processing. However, it is also the case that multimodal cues may be less used by L2 comprehenders because linguistic processing is more demanding than for L1 comprehenders, leaving more limited resources for the processing of multimodal cues. In this study, we investigated how L2 comprehenders use multimodal cues in naturalistic stimuli (while participants watched videos of a speaker), as measured by electrophysiological responses (N400) to words, and whether there are differences between L1 and L2 comprehenders. We found that prosody, gestures, and informative mouth movements each reduced the N400 in L2, indexing easier comprehension. Nevertheless, L2 participants showed weaker effects for each cue compared to L1 comprehenders, with the exception of meaningful gestures and informative mouth movements. These results show that L2 comprehenders focus on specific multimodal cues – meaningful gestures that support meaningful interpretation and mouth movements that enhance the acoustic signal – while using multimodal cues to a lesser extent than L1 comprehenders overall.

## Introduction

In face-to-face communication, language is typically accompanied by a wealth of multimodal cues. These cues span the auditory (such as prosody) and visual (such as gestures and mouth movements) modalities and can provide valuable information to both native (L1) and second (L2) language speakers. Prosodic accentuation, indexed by changes of pitch, intensity, and duration can enhance word recognition by increasing the saliency of specific syllables and emphasizing certain words^1–3^. Similarly, hand gestures, whether meaningful (meaningful gestures, i.e. iconic gestures that describe imagistic properties of a referent, e.g. “draw”—hand mimes holding and moving a pen, and concrete pointing gestures, e.g. “hair”—pointing at the speaker’s hair) or rhythmic (beat gestures, i.e. meaningless gestures time-locked to the speech rhythm^4^), modulate language processing by providing semantic information about the referent^5,6^ or by increasing the saliency of specific words^7,8^. Mouth movements can facilitate word recognition by providing visual information about the pronunciation of words^9–11^. Despite the importance of multimodal cues in face-to-face language comprehension, previous studies have often treated them as peripheral^12,13^, investigating language comprehension via written or listening tasks (in this case, often controlling for prosodic cues). Even when investigating the impact of multimodal information, previous experimental tasks typically manipulated only one cue in isolation (e.g. Ref.^14–16^) while controlling the others. However, such manipulations can result in stimuli that are not ecologically valid, as multimodal cues co-occur with linguistic cues and other multimodal cues in real-world contexts. Thus, for example, mouth movements are always reliably associated with individual words, even across dialectal differences^17^. There is substantial evidence that mouth movements are beneficial to L1 speakers, especially in challenging listening environments, a phenomenon referred to as inverse effectiveness^18–20^. Beat gestures are closely temporally aligned to the prosodic characteristics of speech, typically occurring on a stressed syllable and even influencing the acoustic perception of where a stress is located^21^.

Approximately 50% of the world's population is bilingual^22^. With international migration continuing to grow^23^, a very large number of individuals acquire a second language after their first language at some point in life but only a handful of studies have investigated multimodal comprehension in L2 (e.g. Ref.^14,24–26^). This lack of research is particularly striking given that most language use is face-to-face where there is an abundance of multimodal information. In contrast to L1 comprehenders, L2 comprehenders who have learnt an L2 after their L1 are constrained by their more limited linguistic experience^27^. This makes language processing inherently more taxing for them^28^ and this may reduce the cognitive resources (such as attention and working memory ^29,30^) available for processing non-linguistic communicative cues^16,31,32^. Furthermore, L2 comprehenders may be less familiar with how multimodal cues are used in their L2 (e.g. English is a stress based language while Chinese is tonal; meaningful gestures can be culturally mediated), which can further reduce the impact of multimodal cues on L2 linguistic processing. While these factors may force L2 comprehenders to simply disregard (at least some) multimodal information, it is also plausible that L2 comprehenders would pay more attention to specific multimodal cues which can mitigate difficulties in language processing^24,26,33^ (e.g. meaningful gestures that provide semantic information). Investigating the role of multimodality in naturalistic L2 comprehension is thus an important area of research that can contribute to a better understanding of the mechanisms underlying L2 processing and, potentially, enhance L2 learning and usage in daily life.

In L1, studies investigating multimodal comprehension have shown that comprehenders dynamically adjust the weight of different cues when processing language^34^. A recent EEG study by Zhang and colleagues^34^ used naturally produced audio-visual speech to ensure ecological validity. Rather than artificially manipulating each multimodal cue, they quantified the predictability of each word in the linguistic context (using surprisal, a measure of the predictability of words in context generated by language models). For each word, they further quantified the naturally occurring multimodal information such as prosody, meaningful and beat gestures, and mouth movements. The researchers investigated whether multimodal information, in combination with linguistic predictability, modulated the N400, an event-related-potential (ERP) component peaking negatively ~ 400 ms post stimulus^35^. The N400 has been associated with the processing of word predictability, as words that are less predictable based on linguistic context, either with lower cloze probability^36^, higher surprisal^37,38^, or outright incompatibility with context^39^, induce more negative N400. While the underlying mechanism is still under debate^40–42^, it is generally agreed that the N400 is a bio-marker of comprehension difficulty associated with word predictability (see review in^35^). Zhang and colleagues^34^ also found that more surprising (i.e. less predictable) words induced larger N400 than less surprising words, but – crucially – the effect was modulated by multimodal cues. In particular, higher pitch, meaningful gestures, and informative mouth movements (only when gestures were present) reduced N400, while beat gestures increased it. The N400 was further reduced when meaningful gestures co-occured with higher pitch prosody. Therefore, in naturalistic materials where multiple cues co-occur, L1 comprehenders use these cues and adjust their reliance on specific cues depending on the other available cues (e.g. increasing the reliance on meaningful gestures when it is highlighted by prosody).

Prior research suggests that prosody, gestures, and mouth movements individually support comprehension in L2 comprehenders, although generally less strongly than for L1 comprehenders. For instance, prosodic accentuation enhances comprehenders’ attention to stressed information and facilitates lexical processing in both L1 and L2 comprehenders^1,14,43^. However, L2 comprehenders may be less able to map prosodic information (such as prosodic stress) to aspects of the linguistic context (such as information status)^14,28,31,44^. Indeed, previous studies have shown that L1 comprehenders can better predict upcoming referents based on prosody compared with L2 comprehenders^31,44^.

Meaningful gestures provide semantic information that supports language processing^5,6^. The effects of meaningful gestures on L2 comprehension are mixed and task dependent. It has been shown that L2 comprehenders benefit more from meaningful gestures than L1 comprehenders^24^, particularly for more complex stories^33^. This is especially relevant to less proficient L2 users^45^. Similarly, mismatching meaningful gestures (e.g. saying “drinking” but performing a “drawing” gesture) elicit more negative N400 in L2 than L1 participants^15,46^. However, meaningful gestures improve accuracy to a smaller extent in L2 than L1 participants when recognizing single words embedded in noise^16,32^. These differences may be due to the fact that in discourse it may be easier to extract meaningful information from gestures than from a single degraded word, or because acoustic processing in compromised conditions is so taxing for L2 comprehenders that no resources are left for gestural processing.

Beat gestures increase the saliency of a word^7^ and may improve L1^47^ and L2^25,48^ processing, although the effects are mixed (in both L1^49,50^ and L2^33^). Naturally produced beat gestures (containing continuous movements rather than a single stroke, as in e.g. Ref.^25^) showed no effect on recall of associated sentences in L1 participants and even worsened it in L2 comprehenders^51^.

Lastly, mouth movements facilitate word recognition in L1^52^ and L2 comprehenders^53^. Previous work indicates L2 comprehenders benefit less from mouth movements in tasks involving single word recognition^16,32^. However, L2 comprehenders look more at the mouth area of the speaker compared to L1 comprehenders^26^ during discourse processing, indicating that they search for additional sensory information in longer and more complex materials, where the mouth information is richer and the linguistic processing more challenging.

In summary, L2 comprehension is modulated by a range of multimodal cues^14,24,25,53^. While most studies found smaller benefits in L2 than L1, some studies reported L2 comprehenders being more sensitive to *some* cues such as meaningful gestures^15,24^ and mouth movements^15,26^. Those studies reporting a larger effect of meaningful gestures and mouth movements in L2 typically used longer connected speech^24,26,45^, rather than single words, as stimuli.

As mentioned above, previous studies on multimodal comprehension in L2 have often manipulated individual cues in isolation, limiting the ecological validity of their findings. Given that multimodal cues naturally co-occur in real-world contexts, forcing one cue to be maximally informative while others carry no information may affect comprehension processes and strategies^54^. For example, while less predictable words are more difficult to process when only linguistic information is present^37^, the difficulty is mitigated when multimodal cues are present (as in naturalistic contexts^34^). Similarly, the use of meaningful gestures to disambiguate word meanings is less effective in studies where speakers use both meaningful and grooming gestures^55,56^.

Only a handful of previous studies have investigated the co-occurrence of pairs of multimodal cues in L2 comprehension. One study^16^ asked participants to identify words embedded in noise and accompanied by meaningful gestures, visible mouth movements, or both. Under heavy auditory degradation, L1 users benefited more from the combination of two cues than L2 users. Eye-tracking data^32^ showed that while both L1 and L2 users were drawn to the face area, L2 users fixated more often than L1 on hand gestures. However, only L1 users' gaze patterns predicted comprehension. These findings suggest a trade-off between multimodal cues, with L2 users paying more attention to gestures but being less efficient in using them. One study^25^ investigated the effect of prosodic accentuation and beat gestures on new L2 word learning and found that the combination of both cues led to better memory performance. However, words accompanied by beat gestures but no accentuation (representing an unnatural condition) resulted in worse performance. Thus, the benefit of multimodal cues for L2 comprehenders varies across cue types and experimental setups.

These studies, however, did not consider other co-occurring multimodal cues (Ref.^16,32^ only focused on visual cues^25^ and did not consider mouth movements). Moreover, they broke (for the purpose of experimental manipulation) the natural correlation of different cues (e.g. by manipulating the presence of gesture/mouth movements, while these cues co-occur in naturalistic materials^16,32^; or by forcing prosody and beat gestures to mismatch, while they match in natural speech^25^).

**Figure 1:**
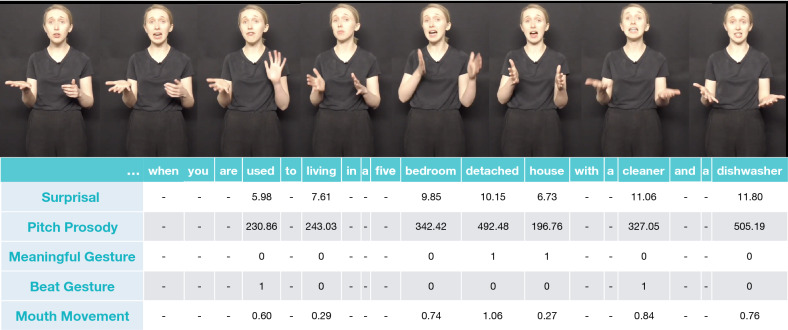
Illustration of the materials and the corresponding annotation. An actress narrated short passages in a naturalistic style and we quantified linguistic (surprisal) and multimodal information (prosody, gestures, and mouth movements) for each content word.

To address these limitations, we conducted an electrophysiological study to investigate: (1) whether L2 comprehenders make use of multimodal information in naturalistic stimuli and (2) how processing differs in L2 compared to L1 comprehenders. Twenty highly proficient non-native English speakers (all native speakers of Mandarin) watched videos of naturalistic audio-visual passages. An actress produced passages chosen from BBC TV scripts with spontaneous prosody, mouth movements and gestures. The actress gave informed consent to use her image in experiments and in open access publications. Following Zhang and colleagues^34^, we quantified linguistic and multimodal information in the naturalistic materials. Specifically, for each content word, we quantified (see Fig. 1): linguistic predictability as surprisal, pitch prosody as averaged pitch per word (mean F0; We refer to this quantification as pitch prosody rather than prosodic accentuation. While accentuated words typically have higher F0, we did not directly manipulate accentuations see also^34^), gestures as meaningful or beat (by manual annotation), and mouth movements according to their informativeness (extracted from a norming study^17^ where L1 participants were asked to guess words just by watching the face of a speaker, thus on mouth movements only, see also^34^). Given that this is the first study investigating whether surprisal affects EEG responses in multimodal L2 comprehension, we first established the exact time window in which surprisal impacted the EEG amplitude by carrying out hierarchical linear modelling (LIMO analysis^57^). Once we had identified the exact time window, we carried out Linear Mixed Effect Regression^58^ (LMER) focusing on the average ERP amplitude. We address our first question – whether L2 comprehenders use multimodal cues in naturalistic materials – by analysing whether the quantification of each cue independently and in interaction modulates the EEG response, and if this manipulation is sensitive to surprisal. We answer our second question – whether and how processing differs in L1 and L2 comprehenders – by comparing the N400 of L2 and L1 participants (taken from Experiment 2 in^34^) watching the same video stimuli.

Overall, we predict that any effect of multimodal cues in L2 will be in the same direction as in L1^25,32,44^ with prosody, meaningful gestures, and mouth movements reducing N400 amplitude as they facilitate processing, and beat gestures inducing larger N400, as they increase the saliency of individual words^34^. We further predict that L2 participants will show an overall smaller effect of multimodal cues on the N400, as in ^14,16^. However, L2 participants may benefit more from some cues such as meaningful gestures^24^ and/or mouth movement^26^, whereas the impact of prosody might be smaller, especially because the prosodic patterns in English greatly differ from prosodic modulation in the participants’ native language (Mandarin, a tonal language).

## Results

**Figure 2:**
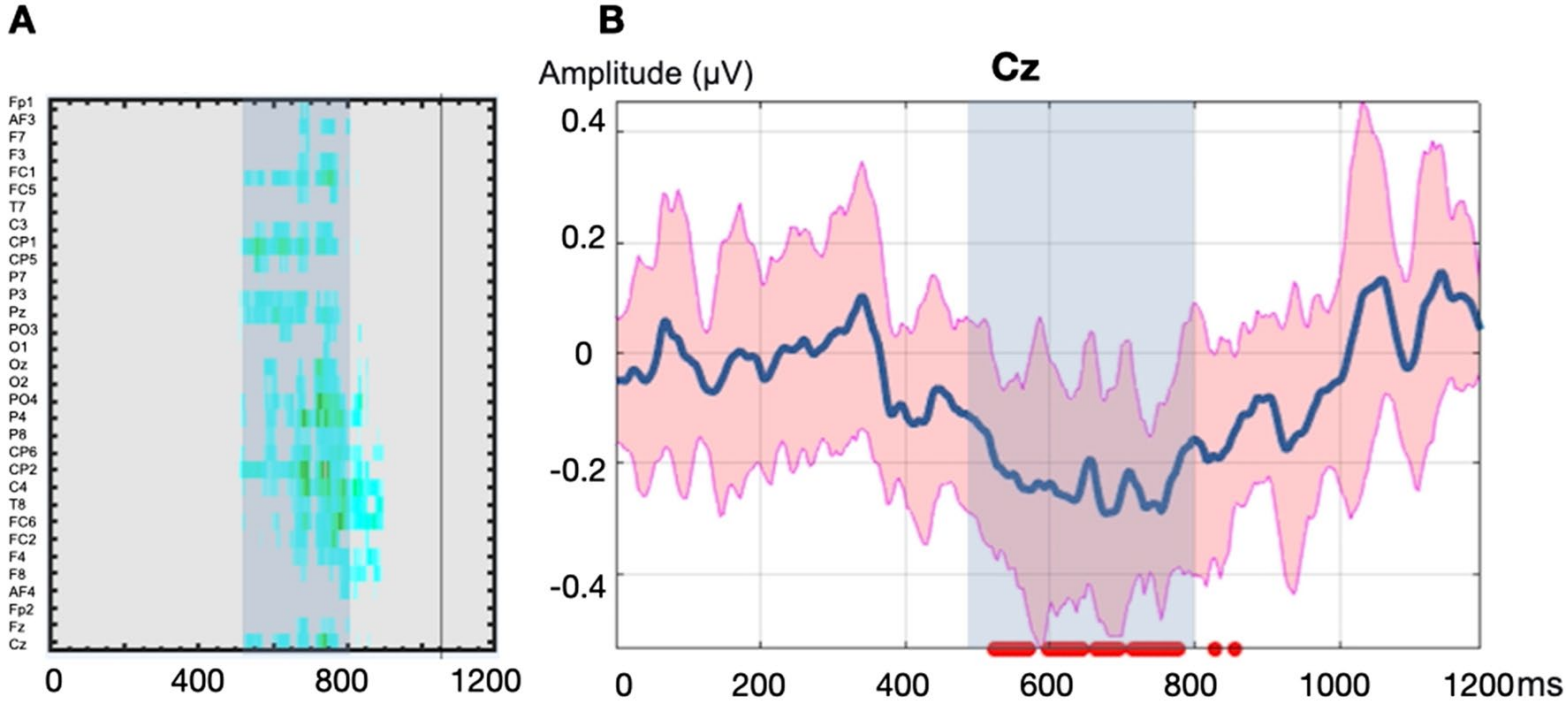
Surprisal modulated the EEG response in the 500–800 ms window (highlighted in blue) for L2 comprehenders. (**A**) Electrodes showed an increased significant negativity in the 500-800 ms window. (**B**) Expected EEG amplitude in the Cz electrode. Surprisal induced more negative EEG signal in ~ 500–800 ms (red area indicates confidence interval; red dotted line under the figure signals significant time window).

First, we used the LIMO toolbox (hierarchical LInear MOdeling)^57^ to establish the time window in which linguistic surprisal mostly affects L2 users. This is a regression-based EEG analysis, which decomposes the ERP signal into a time-series of beta coefficient waveforms associated with a continuous variable, and identifies the significant time window in each electrode. As shown in Fig. 2, words with higher surprisal showed more negative EEG signal in the 500–800 ms post-stimulus time window than words with lower surprisal. While this time window is relatively late, previous studies have reported N400 being later in L2^59^, reflecting greater processing difficulties. Therefore, we focused on the 500–800 ms time window in all following analyses.

### Analysis 1: do multimodal cues modulate L2 processing in naturalistic stimuli?

We treated the mean N400 (within 500–800 ms as noted above) of L2 participants as the dependent variable, and the quantification of multimodal cues and their interactions as independent variables in a LMER analysis. Below, we only report significant effects of multimodal cues and their interactions with surprisal as they are relevant to the research question. A complete overview of the results can be found in the S.M.

**Figure 3:**
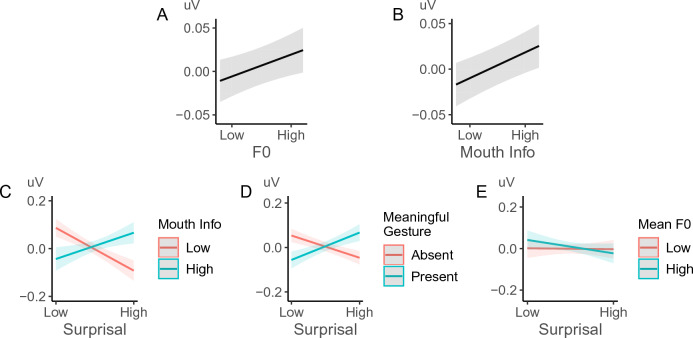
Multimodal cues modulated L2 processing. Higher pitch prosody (**A**) and mouth movements (**B**) induced less negative N400 overall. Furthermore, the impacts of (**C**) mouth movement and (**D**) meaningful gestures were larger for words with higher surprisal, while the impact of (**E**) pitch prosody was larger for words with lower surprisal. The plots show the predicted value of the mean amplitude of the ERP within 500–800 ms (with confidence intervals). See EEG waveforms in S.M.. The same conventions apply to all subsequent figures.

The main effect of surprisal was significant: less predictable words induced a significantly more negative N400 (β = − 0.008, SE = 0.002, p < 0.001). Crucially, multimodal cues also modulated ERP amplitude (Fig. 3): words with higher pitch (β = 0.004, SE = 0.002, p = 0.011) and more informative mouth movements (β = 0.007, SE = 0.001, p < 0.001) elicited a less negative N400. For words that were more surprising in context, the presence of more informative mouth movements (β = 0.010, SE = 0.002, p < 0.001) and meaningful gestures (β = 0.019, SE = 0.001, p < 0.001) reduced the corresponding N400 amplitude. In contrast, the N400 reduction associated with higher pitch was larger for less surprising words (β = − 0.006, SE = 0.002, p < 0.001).

### Analysis 2: do multimodal cues modulate L1 and L2 processing in the same way?

We compared the results from our L2 investigation to those of L1 participants reported in Zhang et al.^34^, Exp.2. The EEG responses (within 500–800 ms) from 20 L1 participants were combined with the newly collected L2 data (note that we found very similar effects when using EEG data from 350 to 850 ms in L1 and 500–800 ms in L2, as well as 400–850 ms for both groups. See S.M. for more details). Native status and interactions between native status and multimodal cues were added to the LMER model used for Analysis 1.

**Figure 4:**
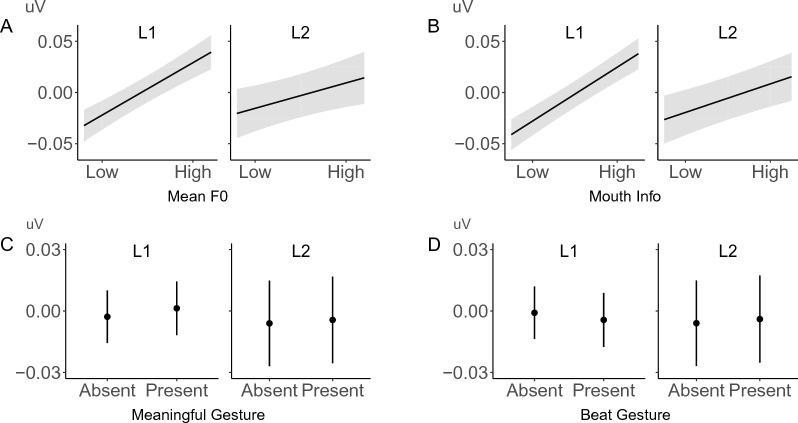
Multimodal cues showed smaller effects in L2. Higher pitch prosody (**A**), more informative mouth movements (**B**), and meaningful gestures (**C**) showed overall smaller N400 reductions in L2 than L1. Beat gestures (**D**) showed a smaller (negative) effect in L2 than L1.

Overall, L2 participants showed smaller effects for all multimodal cues (Fig. 4). Surprisal induced a smaller N400 effect in L2 than L1 (β = − 0.009, SE = 0.001, p < 0.001). In addition, L2 participants also showed a smaller facilitatory effect (indexed by the reduction of N400 amplitude) for each multimodal cue, including pitch (β = 0.004, SE = 0.001, p < 0.001) especially for higher surprisal words (β = 0.003, SE = 0.001, p = 0.007), mouth movements (β = 0.004, SE = 0.001, p < 0.001), and meaningful gestures (β = 0.002, SE = 0.001, p = 0.012). L2 data also showed a smaller increase of the N400 in the presence of beat gestures (β = − 0.006, SE = 0.001, p < 0.001).

**Figure 5:**
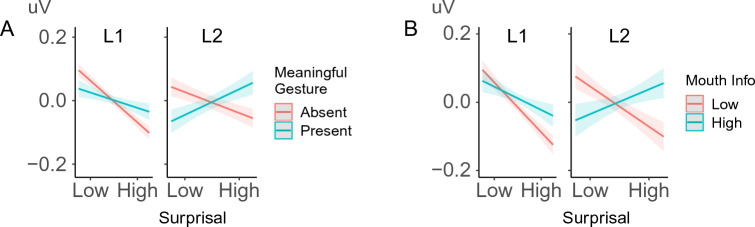
L2 comprehenders showed less negative N400 for less predictable words with meaningful gestures (**A**) and mouth movements (**B**) than L1 comprehenders.

Crucially, despite this general pattern, L2 participants also showed a greater reduction of N400 negativity than L1 speakers for more surprising words when these were accompanied by meaningful gestures (β = − 0.008, SE = 0.001, p < 0.001) or informative mouth movements (β = − 0.007, SE = 0.001, p < 0.001, see Fig. 5).

## Discussion

Our study characterises for the first time how multimodal cues, namely prosody, gestures, and mouth movements, affect comprehension of naturalistic audio-visual materials in L2. We investigated how multimodal cues modulate the N400, an ERP component sensitive to comprehension difficulty associated with word predictability^12,37,38^. We found that highly proficient L2 users were sensitive to the predictability of words in context, as shown by a more negative N400 amplitude for less predictable words. This result is consistent with previous research^59^. Moreover, we demonstrated that multimodal cues modulate predictability. Firstly, words with higher pitch induced less negative N400 overall, particularly for more predictable words. Secondly, informative mouth movements and meaningful gestures also elicited less negative N400, particularly for less predictable words. These findings indicate that L2 users dynamically incorporate multimodal cues during face-to-face comprehension based on context.

Broadly, these effects are similar to what Zhang et al. observed in L1^34^ with higher pitch, meaningful gestures, and informative mouth movements inducing less negative N400. Overall, L2 comprehenders benefited less from multimodal cues than L1 comprehenders, as indicated by the smaller N400 reduction. However, this was not always the case: when words were less predictable based on their linguistic context, L2 users benefited more from two cues: meaningful gestures and informative mouth movements.

### L2 comprehenders benefit from multimodal information in naturalistic context

In line with prior behavioural studies^14,43^, we found that higher pitch facilitates L2 comprehension. However, this effect was limited to more predictable words. Previous research has suggested that L2 users are less capable of integrating prosodic and linguistic information^14,31,44^, potentially due to difficulties in extracting prosodic information^27^ and/or integrating it across channels during online processing of connected speech^28,60^. This may be especially the case for typologically different languages like English (stress-based language^61^) and Mandarin (tone-based language^62^). It could also be the case that less predictable words are simply less known to L2 comprehenders and therefore drawing attention to their phonological form might not have been sufficient to enhance their processing.

Furthermore, our findings suggest that meaningful gestures have a facilitatory effect on L2 comprehension. While previous behavioural^24,45^ and electrophysiological studies^15,46^ have reported that L2 comprehenders are sensitive to gestural information, our study showed that naturally occurring congruent meaningful gestures facilitate comprehension. This facilitatory effect is especially strong for more surprising words, possibly because the visual channel's semantic information can supplement or replace the linguistic information in the input.

In addition, we report, for the first time, that informative mouth movements also reduce N400 amplitudes in L2 comprehension. As mentioned in the introduction, it is well established that observing mouth movements can improve word recognition in a noisy environment^16,32^. We further show that L2 comprehenders benefit from informative mouth movements in clear and naturalistic speech, as mouth movements facilitate the recognition of less expected words. This effect was not found by Zhang and colleagues in L1 comprehenders^34^, who did not manipulate speech clarity.

### Less but smarter: L2 comprehenders weigh multimodal cues differently compared to L1

Consistent with previous studies (prosody^14,31,44^; gestures^16,32,63^; mouth^16,63^), L2 comprehenders use multimodal cues similarly to L1 comprehenders overall, although the effect of each cue is smaller. It is possible that L2 processing is more demanding, therefore fewer resources are left for processing multimodal cues. For example, a previous study^64^ reported that while seeing a speaker facilitates spoken word recognition in noisy environments, it also increases cognitive load and therefore listening effort. Alternatively, L2 users may be less familiar with the patterns of multimodal cues in the non-native language, and therefore less efficient in extracting multimodal information while processing linguistic content. For example, it has been found that Turkish and English speakers produce different meaningful gestures for the same events, in accordance with the syntactic structures of their native languages^65^. As frequent gestures can induce a larger facilitatory effect to comprehension than less frequent ones^66^, L2 comprehenders may benefit less from non-native gestures in general as they are less familiar with them.

However, our study found that when words are less predictable based on the available linguistic information, L2 users benefit more than L1 users from two visual cues, namely meaningful gestures and informative mouth movements. Compared with prosody and beat gestures (that may show more distinct cross-linguistic patterns and smaller effects in L2 than in L1 across the board), meaningful gestures and mouth movements provide clear semantic or sensory information that can aid linguistic processes. This contrast suggests that L2 users may be able to regulate (automatically or strategically) cognitive resources dynamically and efficiently, by assigning more weight to informative multimodal cues when linguistic information is difficult, and reducing the weight of multimodal cues when they are less useful. Note here that these results refer to bilinguals for whom one language (L1) is clearly dominant and may not generalise to bilinguals for whom both languages are well established (e.g. bilinguals from birth who live in bilingual communities).

### Towards a multimodal theory of L2 processing

Our study provides evidence that challenges existing theories of language processing, including in L2 comprehenders. Traditional mechanistic theories of L1 comprehension have proposed multiple mechanisms that single out linguistic processing, often operating at distinct levels of representation (e.g. lexical access, reanalysis, binding, unification^67–69^). However, emerging findings on multimodal language comprehension raise questions about how these theoretical frameworks could incorporate processing of multimodal cues. For instance, it has been proposed that linguistic and multimodal cue information may be processed in an encapsulated manner, and therefore that multimodal cues would be considered and combined with linguistic processing only later in time^13,70,71^ and as a supplementary source of information that may support comprehension. However, our results suggest that multimodal cues are already integrated with linguistic predictability during the N400 time window. Thus, our findings call for an account of language comprehension where the processing of linguistic and multimodal input takes place in a more interactive than modular fashion.

Our data suggest a potential mechanism where linguistic and multimodal information is dynamically weighted and orchestrated by the brain according to prior context. This is in line with the cue integration approach of language processing, which postulates that the brain takes into consideration all possible cues and combines them in a weighted manner to form linguistic or event representations (e.g. ^72^). The weight, or interchangeably the reliability of each cue, is reliant on recent instances of its use. New multimodal frameworks can also accommodate our findings. For instance, Holler and Levinson^73^ propose that multimodal cues are bonded together as a gestalt and dynamically modulate language processing via different mechanisms. Skipper^74^ further proposed a neurobiological theory in which linguistic and multimodal information is processed in different but partially overlapping sub-networks that constantly communicate with each other. However, while these theories are broadly in line with our results, they are still underspecified and cannot predict individual findings from our studies.

Furthermore, there has been no theory about cue integration explicitly devoted to L2 processing. Studies on L2 processing have noted the impact of limited cognitive resources, such as attention and working memory, on the speed and availability of resources in non-native language comprehension (e.g. ^29,30^). Some theories have also proposed that L2 comprehenders encounter larger processing difficulties when mapping information across different representation domains (interface hypothesis^60^), such as linguistic and non-linguistic domains. Our results provide further support for such accounts by showing that the processing of multimodal cues is reduced in general. However, our findings also highlight that meaningful gestures and mouth movements can function to facilitate comprehension in L2 comprehenders. Therefore, our study calls for revisions and specifications of theories of L2 processing to accommodate the dynamic adjustment and the associated cognitive constraints.

To conclude, our study provides the first electrophysiological investigation of L2 processing in more naturalistic materials where more than one cue co-occurs. We characterised how multimodal cues jointly modulate L2 comprehension, and highlighted those cues that can be most useful for L2 comprehenders (i.e. meaningful gestures and mouth movements). Our findings point to the need for a broader experimental and theoretical focus in investigating L2 processing: as our results clearly show, L2 comprehenders always use multimodal cues that occur in naturalistic materials, and actively and efficiently weight these based on the multimodal context.

## Methods

### Participants

Twenty (16 females, aged 18–40) students were recruited from University College London. All participants were highly proficient L2 English speakers (Mandarin-English; > 7.5/9 in IELTS listening tests; > 2 years in an English-speaking country; use English daily). All participants had normal hearing, vision, and no known neurological disorder. All participants gave written informed consent and were paid £7.5/hour for participation. All experimental protocols were approved by the UCL Research Ethics Committee and all procedures were performed in accordance with relevant guidelines and regulations.

### Materials

Materials were taken from Experiment 2 by Zhang and colleagues^34^ (see Fig. 1 for an example). They included 83 passages from the BBC collection (https://www.bbc.co.uk/writersroom/scripts). Forty-two native English speakers rated the chosen passages on gesturability (i.e., how easy it is to gesture, on a Likert scale from 0 to 5) as well as whether the sentence was meaningful and grammatically acceptable. All the passages used in the study had 1) a mean gesturability score above 2; 2) a grammaticality judgment above 70%; and 3) a meaningfulness score higher than 70%. 79 of the passages were used as experimental stimuli and 4 was used as practice trials.

The passages were then produced by a native English-speaking actress with natural prosody and facial expressions. Two versions (with and without gestures) were recorded (duration 10 s-34 s). For the videos with gestures, the actress was asked to produce meaningful gestures, but with no specific instruction on which gesture to make or how often the gestures should be produced. For the videos without gestures, the actress was asked to stand still with her arms alongside the body.

Participants rated the difficulty of each passage after the experiment on a 1–5 scale to determine whether any stimulus was too difficult to understand. The average difficulty score of the 79 passages was not significantly different across L1 participants from Zhang et al., 2021 and L2 participants from the current study (L1: M = 2.53 ± 0.53; L2: M = 2.58 ± 0.76; paired-sample *t* test p = 0.46), with all values staying within ± 3 standard deviations, which indicated that all 79 stimuli in our study were sufficiently easy to understand for all the L2 participants and, consequently, can be included in the following analyses.

### Procedure

Participants sat ~ 1 m facing the computer screen and wore earphones. After four practice trials, participants were presented with 79 video clips (the presentation of gesture/no-gesture was randomized and counterbalanced across participants). Videos were displayed with an inter-trial interval of 1000 ms. Forty videos were followed by yes/no comprehension questions to ensure that participants paid attention to the stimuli (mean accuracy = 0.82, p < 0.001 in one sample t-test comparing against chance level). Participants were instructed to watch the videos carefully and answer the questions as quickly and accurately as possible. See an example below:

**Passage:** “We’d literally just moved in, we were still living out of boxes. It was dark, I hadn’t sorted out the wiring yet. She was at the top of the stairs and… They said she must have fallen awkwardly.

**Question:** “Did the girl mentioned by the speaker fall down the stairs?”

The experimental session was divided into four blocks and participants were allowed to take a break between each block. A 32-channel BioSimi system was employed for EEG data collection using Ag/AgCl electrodes with 24-bit resolution, following the 10–10 international system layout. The CMS and DRL electrodes served as the common reference. Elastic head caps ensured electrode stability. Additional external electrodes were affixed to the left/right mastoids for offline reference, and two more on the lower left eye and right canthus to monitor blinks and eye movements. Electrolyte gel enhanced connectivity. Electrode offsets were maintained within ± 25 mV to assess relative impedance differences. Recordings took place in a temperature-controlled (18 °C) shielded room. Participants were asked to avoid moving, keep their facial muscles relaxed, and reduce blinking. The whole EEG experimental session lasted approximately 60 min.

### Quantification of cues

For each video, we followed Zhang and colleagues in annotating the onset and offset of each word (mean duration = 508 ms, SD = 306), and then quantified the informativeness of each cue per content word (i.e. nouns, adjectives, verbs and adverbs) as below^34^.

#### Surprisal

(mean surprisal = 8.17, SD = 1.92) was obtained using a bigram language model trained on the 1st slice of the ENCOW14-AX corpus^75^. We then calculated the surprisal score of each word based on all previous content words using the following formula:

$${\text{Surprisal}}\left( {{\text{W}}_{{\text{t}}} } \right) \, = \, - {\text{log}}P\left( {{\text{W}}_{{\text{t}}} |{\text{W}}_{{{1} \ldots {\text{t}} - {1}}} } \right).$$

Here, W indicates the current word, and W_1…t-1_ stands for the previous content words in the passage.

#### Prosody

Was quantified as the mean F0 per word (mean F0 = 288 Hz, SD = 88), extracted using Praat.

#### Gestures

Were coded as meaningful gestures or beat gestures by two expert coders (reliability coding was carried out by a third coder; intercoder reliability > 95%, kappa > 0.90, p < 0.001). Meaningful gestures (N = 457) included iconic gestures (e.g. drawing movements for the word “drawing”) and deictic gestures (e.g. pointing to the hair for “hair”). Beat gestures (N = 340) comprised rhythmic hand movements without clear meaning. Each word was then linked either to a meaningful gesture (if a meaningful gesture associated with its meaning is present), a beat gesture (if a beat gesture overlapped with it), or no gesture.

#### Mouth informativeness

Was taken from the mouth and facial informativeness norms^17^ (mean informativeness = − 0.67, SD = − 0.29). In the norming study, 150 L1 English speakers were presented with a muted video-clip of an actress (native English speaker) producing content words individually. Participants were asked to watch the muted videos and guess the words based exclusively on mouth movement. The averaged phonological distance between the guesses and the answers was then calculated using the PanPhon Python package^76^^.^ The distance is then multiplied by –1 to produce the mouth and informativeness score, so that a larger value represents higher informativeness (maximum = 0).

### Pre-processing of EEG data

The data was pre-processed using EEGLAB (version 14.1.1) and ERPLAB (version 7.0.0) running under MATLAB (R2017b). All electrodes were included. Triggers were sent at the onset of each video, and the word onset was subsequently calculated from the word boundary annotation. Any lag between the presentation of the trigger and the stimuli was also measured and corrected (mean = 40.03 ms, SD = 1.68). EEG files were referenced to the mastoids, down-sampled to 256 Hz, separated into − 100 to 1200 ms epochs time-locked to the onset of each word, and filtered with a 0.05–100 Hz band-pass filter. Artifacts (e.g. blinks and muscle noise) were first corrected with Independent Component Analysis (ICA), and the remaining artifacts were rejected using a moving-window peak-to-peak analysis (voltage threshold = 100 µV, moving-window full width = 200 ms, window step = 20 ms) and step-like artifact analysis (voltage threshold = 35 µV, moving window full width = 400 ms, window step = 10 ms). This resulted in an average rejection rate of 8.69% of the overall data. Then, an additional 30 Hz low-pass filter was applied to the data to further reduce high-frequency noise. Due to the likely overlap between any baseline period (− 100 to 0 ms) and the EEG signal elicited by the previous word, we did not perform a baseline correction, but instead extracted the mean EEG amplitude in this time interval and later used it as a control variable in the analysis^34,37^.

### Hierarchical linear modelling analysis

Rather than specifying an N400 window a priori, we established the precise time window in which linguistic surprisal has an effect^34^ using the LIMO toolbox (hierarchical LInear MOdeling)^57^. This regression-based ERP analysis linearly decomposes an ERP into time-series of beta coefficient waveforms elicited by continuous variables. Significant differences between the beta coefficient waveforms and zero (flat line) indicate that a variable elicited a significant effect in a certain time window, as the increase/decrease of the variable induces higher/lower EEG response. In the first level analysis for each participant, a regression was performed for each data point in the 0–1200 ms time window per electrode and per word, with EEG voltage as the dependent variable and word surprisal as the independent variable. In this way we could detect when surprisal had a significant effect for each participant. In the second level analysis we compared the beta matrix resulting from the first level analysis against 0 using a one-sample *t* test (bootstrap set at 1000, clustering corrected against spatial and temporal multiple comparison).

### Linear mixed effect regression analysis (LMER)

We conducted all our statistical analyses using LMER from the lme4 package^58^ under RStudio (version 4.0.4). As the dependent variable, we used the ERPs associated with each content word (without baseline correction) that we extracted from 32 electrodes in a 500–800 ms time-window (as identified in the LIMO analysis). Mean ERPs in the time window of − 100 to 0 ms were extracted as well as a baseline. Following Zhang and colleagues^34^, we compared the same words across the with/without gesture videos, instead of comparing different words (with and without gesture) in the gesture videos only. This is because words likely to be accompanied by meaningful gestures (e.g. “combing”) are semantically very different from words that are not (e.g. “pleasing”). Thus, for all the models below, we only include the words with gestures (from videos with gestures) and the corresponding words without gesture (from videos without gestures) to balance the number of observations between groups.

#### Analysis 1: how do multimodal cues affect L2 processing?

Here we analysed how multimodal cues interact to affect the N400 of L2 participants. The independent variables included in the analysis were: (1) main effects of: surprisal, mean F0, meaningful gesture, beat gesture, mouth movements; (2) two-way interactions between these cues; (3) three-way interactions involving surprisal and any two multimodal cues; and (4) control variables including baseline (− 100 to 0 ms), word length, word order in the passage, passage order in the experiment, and x, y, z coordinates of each electrode. All electrodes were included with coordinates as control following Zhang and colleagues^34^ to allow for more standardised comparison (as L1 and L2 participants may show different scalp distribution). No main or interaction effects showed multicollinearity, with variance inflation factor (VIF) lower than 2.4, and kappa = 5.63. All continuous variables, including ERP, surprisal, mean F0, mouth informativeness, baseline, word length, word order, sentence order and x, y, z position of electrodes were standardised (centred and scaled) so that each coefficient represents the effect size of the variable. Surprisal was log-transformed to normalize the data. All categorical variables were sum-coded so that each coefficient represents the size of the contrast from the given predictor value and the grand mean (intercept). We further included the highest interactions (three-way interactions between surprisal and cues) as random slopes for participants^77^. We did not include lemma as random intercept or other interactions as random slopes due to convergence issues.

#### Analysis 2: do multimodal cues show the same effects in L1 and L2?

Here we compared results from L2 participants to those of L1 participants who were tested with the same materials from Experiment 2 by Zhang and colleagues^34^. The EEG responses within 500–800 ms from the 20 L1 participants were combined with the L2 data described above (as 500–800 ms did not cover the full N400 window for L1 participants, we also compared 350–850 ms for L1 with 500–800 ms for L2 participants. See S.I. for full results). Native status and the interaction between native status and the multimodal cues were added to the LMER model presented in Analysis 1. No main effect or interaction showed multicollinearity (VIF < 2.5, kappa = 5.76).

### Supplementary Information

Supplementary Information.

## Data Availability

Data and script can be found on OSF https://osf.io/zk47n/?view_only=e7d847fab90945c5bfd69dc1a59dc887.
