# Supplementary material for: The role of multimodal cues in second language comprehension
Source: Sci Rep. 2023 Nov 27;13:20824. doi: 10.1038/s41598-023-47643-2 (PMC10682458; doi:10.1038/s41598-023-47643-2)
Supplement: Supplementary file 1 — Supplementary Information. [file 41598_2023_47643_MOESM1_ESM.pdf]

Supplementary Materials for

The Role of Multimodal Cues in Second Language Comprehension

Ye Zhang[1], Rong Ding[2], Diego Frassinelli[3], Jyrki Tuomainen[4], Sebastian Klavinskis-Whiting[5],

Gabriella Vigliocco[1]

[1]Experimental Psychology, University College London, UK

[2]Language and Computation in Neural Systems, Max Planck Institute for Psycholinguistics, Netherlands

[3]Department of Linguistics, University of Konstanz, Germany

[4]Speech, Hearing and Phonetic Sciences, University College London, UK

[5]Christ Church College, Oxford

Corresponding Author: Ye Zhang (y.zhang.16@ucl.ac.uk)

Table of contents

1. LMER Analysis 2
2. Full results and plots 3
3. Selection of Time Window 13
4. Full stimuli 20

1. LMER Analysis

1) Exclusion criteria

We excluded from the analyses: (a) content words without a surprisal value. Very few words do not have any surprisal value (n=13), due to the lack of co- occurrence between this word and its context in the training corpus. (b) Words without a mean F0 score. Very few words (n=2) showed pitch error when using Praat to automatically extract pitch (e.g. when the vowel is pronounced very quietly). c) Words with both meaningful and beat gesture (n=6). These instances usually represent the speaker producing a meaningful gesture but then a quick beat gesture for a word, or using one hand to produce a meaningful gesture but the other to produce a beat. Given the rarity of this phenomenon, we excluded them from the analysis thus removing any interaction between meaningful and beat gestures. d) Words occurring without any gesture in the "with gesture" condition, and the corresponding words in without gesture videos (n=685). This is to reduce the imbalance of the data, otherwise the without gesture condition would include not only the corresponding without gesture words of all with gesture words, but also all words in d), making this group ~3 times larger than the with gesture one. We compared the same item across the with/without gesture videos, instead of with or without gesture words in the with gesture videos only (different items). This is because words likely to be accompanied by meaningful gestures (e.g., *combing*) are semantically very different from words that are not (e.g., *pleasing*). Thus, comparison of the same words produces clearer results.

2) Fixed and random variables

We originally included frequency (derived from the ENCOW corpus) as control variable. However, frequency is removed from the final model due to multiple collinearity with surprisal, as both measures capture linguistic probability to different extents.

The word order variable (in the control variables) is identified as the position of the current word in the sentence. E.g. in "Emma screamed …", word order of "scream" is 2.

For each word, its lemma is extracted (e.g. lemma "work" includes both word "worked" and "working") and subsequently included as random intercept. Therefore, the model assumes each lemma may have a different intercept. The inclusion of by participant and item intercept (and slope when possible) is the traditional setup for such models when dealing with psycholinguistic studies (Barr et al., 2013).

3) Model formulas

The model formula of Analysis 1 is:

```
N400~ surprisal + meanF0 + mouthInfo + meaningfulGesture + beatGesture +
surprisal:meanF0 + surprisal:mouthInfo + surprisal:meaningfulGesture +
surprisal:beatGesture +
meanF0:mouthInfo + meanF0:meaningfulGesture + meanF0:beatGesture +
mouthInfo:meaningfulGesture + mouthInfo:beatGesture+
```

```
surprisal:meanF0:mouthInfo + surprisal:meanF0:meaningfulGesture +
surprisal:meanF0:beatGesture + surprisal:meaningfulGesture:mouthInfo +
surprisal:beatGesture:mouthInfo +
wordOrder + wordLen + sentenceOrder + baseline + x + y + z +
(1+surprisal:meanF0:mouthInfo+ surprisal:meanF0:meaningfulGesture
+surprisal:meanF0:beatGesture +surprisal:meaningfulGesture:mouthInfo
+surprisal:beatGesture:mouthInfo |participantID)
```

The model formula of Analysis 2 is:

```
N400~ surprisal + meanF0 + mouthInfo + meaningfulGesture + beatGesture +
surprisal:meanF0 + surprisal:mouthInfo + surprisal:meaningfulGesture +
surprisal:beatGesture +
meanF0:mouthInfo + meanF0:meaningfulGesture + meanF0:beatGesture +
mouthInfo:meaningfulGesture + mouthInfo:beatGesture+
surprisal:meanF0:mouthInfo + surprisal:meanF0:meaningfulGesture +
surprisal:meanF0:beatGesture + surprisal:meaningfulGesture:mouthInfo +
surprisal:beatGesture:mouthInfo +
surprisal:isNative + meanF0:isNative + mouthInfo:isNative +
meaningfulGesture:isNative + beatGesture:isNative +
surprisal:meanF0:isNative + surprisal:mouthInfo:isNative +
surprisal:meaningfulGesture:isNative + surprisal:beatGesture:isNative +
meanF0:mouthInfo:isNative + meanF0:meaningfulGesture:isNative +
meanF0:beatGesture:isNative +
mouthInfo:meaningfulGesture:isNative + mouthInfo:beatGesture+
surprisal:meanF0:mouthInfo:isNative +
surprisal:meanF0:meaningfulGesture:isNative +
surprisal:meanF0:beatGesture:isNative +
surprisal:meaningfulGesture:mouthInfo:isNative +
surprisal:beatGesture:mouthInfo:isNative +
wordOrder + wordLen + sentenceOrder + baseline + x + y + z +
(1+surprisal:meanF0:mouthInfo+ surprisal:meanF0:meaningfulGesture
+surprisal:meanF0:beatGesture +surprisal:meaningfulGesture:mouthInfo
+surprisal:beatGesture:mouthInfo |participantID)
```

---

2. *Full results and plots*

1) Analysis 1

See Table S1 below for the full results of Analysis 1 assessing the impact of multimodal cues on N400 in L2 group.

Table S1. *Full result: LMER model on N400 (500-800ms) for L2 group.*

**Fixed Effects**	**β**	**Std Error**	**t**	**p**
(Intercept)	0.004	0.011	0.341	0.736
**Predictor Variables**				
Surprisal	-0.008	0.002	-5.191	<.001***
Mean F0	0.004	0.002	2.535	0.011*
Mouth Informativeness	0.007	0.001	4.957	<.001***

Meaningful Gesture (Present)	0.002	0.001	1.483	0.138
Beat Gesture (Present)	0.002	0.001	1.552	0.121
Surprisal:Mean F0	-0.006	0.002	-3.526	<.001***
Surprisal:Mouth Informativeness	0.010	0.002	6.186	<.001***
Surprisal:Meaningful Gesture (Present)	0.019	0.001	15.151	<.001***
Surprisal:Beat Gesture (Present)	0.001	0.001	0.447	0.655
Mean F0:Mouth Informativeness	-0.003	0.001	-2.604	0.009**
Mean F0:Meaningful Gesture (Present)	0.003	0.001	3.067	0.002**
Mean F0:Beat Gesture (Present)	0.001	0.001	0.640	0.522
Mouth Informativeness:Meaningful Gesture (Present)	0.004	0.001	3.549	<.001***
Mouth Informativeness:Beat Gesture (Present)	-0.006	0.001	-4.400	<.001***
Surprisal:Mean F0:Mouth Informativeness	-0.002	0.006	-0.419	0.680
Surprisal:Mean F0:Meaningful Gesture (Present)	0.012	0.005	2.323	0.031*
Surprisal:Mean F0:Beat Gesture (Present)	0.000	0.005	-0.053	0.958
Surprisal:Mouth Informativeness:Meaningful Gesture (Present)	0.007	0.004	1.783	0.089
Surprisal:Mouth Informativeness:Beat Gesture (Present)	0.003	0.005	0.501	0.621
**Control Variables**				
Word Order	-0.002	0.001	-2.031	0.042*
Word Length	0.007	0.001	6.291	<.001
Sentence Order	-0.002	0.001	-1.766	0.077
Baseline	0.759	0.001	768.112	<.001***
Electrode X	-0.006	0.001	-5.954	<.001***
Electrode Y	0.003	0.001	2.810	0.005**
Electrode Z	-0.004	0.001	-4.399	<.001

**Random Effects**		**Variance**	**Std.Dev.**
Participant ID	(Intercept)	0.002	0.048
	Surprisal:Mean F0:Mouth Informativeness	0.001	0.024
	Surprisal:Mean F0:Meaningful Gesture (Present)	0.000	0.022

Surprisal:Mean F0:Beat Gesture (Present)	0.001	0.023
Surprisal:Mouth Informativeness:Meaningful Gesture (Present)	0.000	0.016
Surprisal:Mouth Informativeness:Beat Gesture (Present)	0.001	0.023

**Model**				
AIC	BIC	logLik	deviance	df.resid
892325	892864	-446113	892227	448591

Notes. * $p < .05$, ** $p < .01$, *** $p < .001$

2) Analysis 2

See Table S2 below for full results of Analysis 2 comparing the impact of multimodal cues in L2 with L1 group.

Table S2. *Full result: LMER model on N400 (500-800ms) for L1 and L2 group.*

**Fixed Effects**	**β**	**Std Error**	**t**	**p**
(Intercept)	-0.002	0.007	-0.300	0.766
**Predictor Variables (effects of cues)**				
Native Status (Native)	-0.003	0.007	-0.407	0.686
Surprisal	-0.016	0.001	-13.866	<.001***
Mean F0	0.008	0.001	7.188	<.001***
Mouth Informativeness	0.011	0.001	10.614	<.001***
Meaningful Gesture (Present)	0.004	0.001	4.977	<.001***
Beat Gesture (Present)	-0.004	0.001	-3.715	<.001***
Surprisal:Mean F0	-0.003	0.001	-2.098	0.036*
Surprisal:Mouth Informativeness	0.003	0.001	3.158	0.002**
Surprisal:Meaningful Gesture (Present)	0.010	0.001	11.340	<.001***
Surprisal:Beat Gesture (Present)	-0.001	0.001	-1.015	0.310
Mean F0:Mouth Informativeness	-0.002	0.001	-3.118	0.002**
Mean F0:Meaningful Gesture (Present)	0.004	0.001	4.981	<.001***
Mean F0:Beat Gesture (Present)	0.006	0.001	5.843	<.001***

Mouth Informativeness:Meaningful Gesture (Present)	0.007	0.001	8.355	<.001***
Mouth Informativeness:Beat Gesture (Present)	0.001	0.001	1.000	0.317
Surprisal:Mean F0:Mouth Informativeness	-0.005	0.004	-1.266	0.213
Surprisal:Mean F0:Meaningful Gesture (Present)	0.003	0.003	0.887	0.380
Surprisal:Mean F0:Beat Gesture (Present)	-0.002	0.004	-0.405	0.688
Surprisal:Mouth Informativeness:Meaningful Gesture (Present)	0.001	0.003	0.256	0.800
Surprisal:Mouth Informativeness:Beat Gesture (Present)	0.004	0.004	1.140	0.261
**Predictor Variables (interactions with native status)**				
Native Status (Native):Surprisal	-0.009	0.001	-8.349	<.001***
Native Status (Native):Mean F0	0.004	0.001	3.586	<.001***
Native Status (Native):Mouth Informativeness	0.004	0.001	4.219	<.001***
Native Status (Native):Meaningful Gesture (Present)	0.002	0.001	2.800	0.005**
Native Status (Native):Beat Gesture (Present)	-0.006	0.001	-5.952	<.001***
Native Status (Native):Surprisal:Mean F0	0.003	0.001	2.683	0.007**
Native Status (Native):Surprisal:Mouth Informativeness	-0.007	0.001	-6.090	<.001***
Native Status (Native):Surprisal:Meaningful Gesture (Present)	-0.008	0.001	-8.644	<.001***
Native Status (Native):Surprisal:Beat Gesture (Present)	-0.002	0.001	-1.584	0.113
Native Status (Native):Mean F0:Mouth Informativeness	0.000	0.001	0.631	0.528
Native Status (Native):Mean F0:Meaningful Gesture (Present)	0.001	0.001	1.325	0.185

Native Status (Native):Mean F0:Beat Gesture (Present)	0.005	0.001	4.481	<.001***
Native Status (Native):Mouth Informativeness:Meaningful Gesture (Present)	0.003	0.001	3.619	<.001***
Native Status (Native):Mouth Informativeness:Beat Gesture (Present)	0.006	0.001	6.805	<.001***
Native Status (Native):Surprisal:Mean F0:Mouth Informativeness	-0.002	0.004	-0.593	0.557
Native Status (Native):Surprisal:Mean F0:Meaningful Gesture (Present)	-0.009	0.003	-2.562	0.014*
Native Status (Native):Surprisal:Mean F0:Beat Gesture (Present)	-0.001	0.004	-0.300	0.765
Native Status (Native):Surprisal:Mouth Informativeness:Meaningful Gesture (Present)	-0.006	0.003	-2.313	0.026*
Native Status (Native):Surprisal:Mouth Informativeness:Beat Gesture (Present)	0.002	0.004	0.447	0.657
**Control Variables**				
Word Order	0.002	0.001	3.128	0.002**
Word Length	0.005	0.001	5.624	<.001***
Sentence Order	0.000	0.001	-0.551	0.582
Baseline	0.746	0.001	1045.731	<.001***
Electrode X	-0.006	0.001	-8.907	<.001***
Electrode Y	0.002	0.001	3.002	0.003**
Electrode Z	-0.004	0.001	-6.200	<.001***

**Random Effects**		**Variance**	**Std.Dev.**
Participant ID	(Intercept)	0.002	0.041
	Surprisal:Mean F0:Mouth Informativeness	0.000	0.022
	Surprisal:Mean F0:Meaningful Gesture (Present)	0.000	0.021
	Surprisal:Mean F0:Beat Gesture (Present)	0.001	0.025
	Surprisal:Mouth Informativeness:Meaningful Gesture (Present)	0.000	0.016

	Surprisal:Mouth Informativeness:Beat Gesture (Present)		0.001	0.023

Model				
AIC	BIC	logLik	deviance	df.resid
1789613	1790419	-894737	1789475	883515

Notes. * $p < .05$, ** $p < .01$, *** $p < .001$

3) EEG plots

We present below the EEG plots of all target variables and up to two-way interactions for L2 and L1 groups (Figure S1-3). For illustration only, continuous variables (surprisal, F0, mouth informativeness) were categorised into high and low groups, each containing 30% highest or lowest values. ERP data was additionally filtered with 15Hz low-pass filter for illustration. The N400 time window (500-800ms) was highlighted. Results of statistical analysis was shown under the group label in grey. The values under L2 represents the significance of a variable in L2 group (Analysis 1), and the values under L1 represents the interaction between this variable and native status (Analysis 2). MG represents meaningful gestures and BG represents beat gestures.

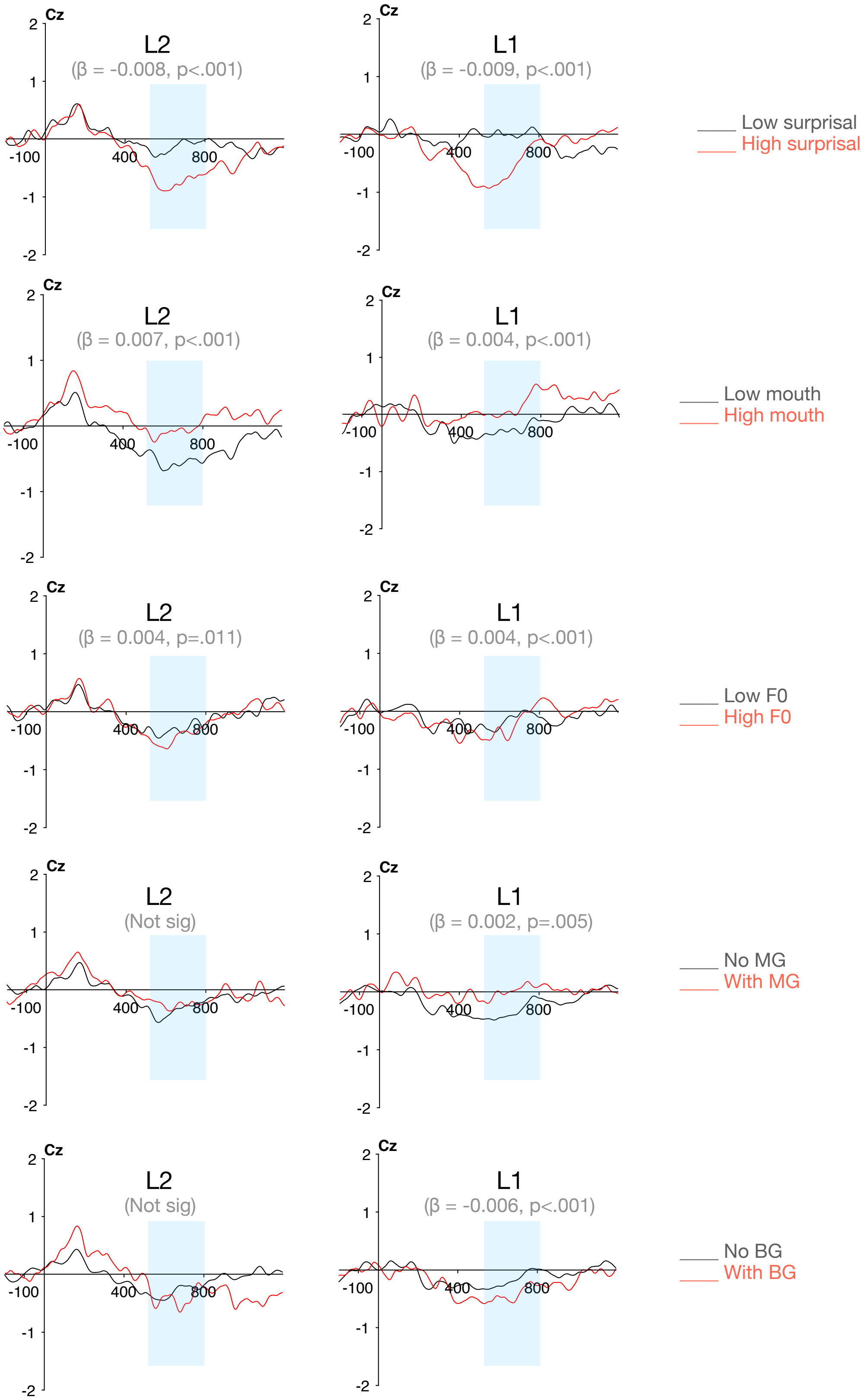

Figure S1. Main effects of surprisal and multimodal cues in L1 and L2. High mouth informativeness and pitch prosody is associated with less negative N400 in L2. All effects were larger in L1 than L2.

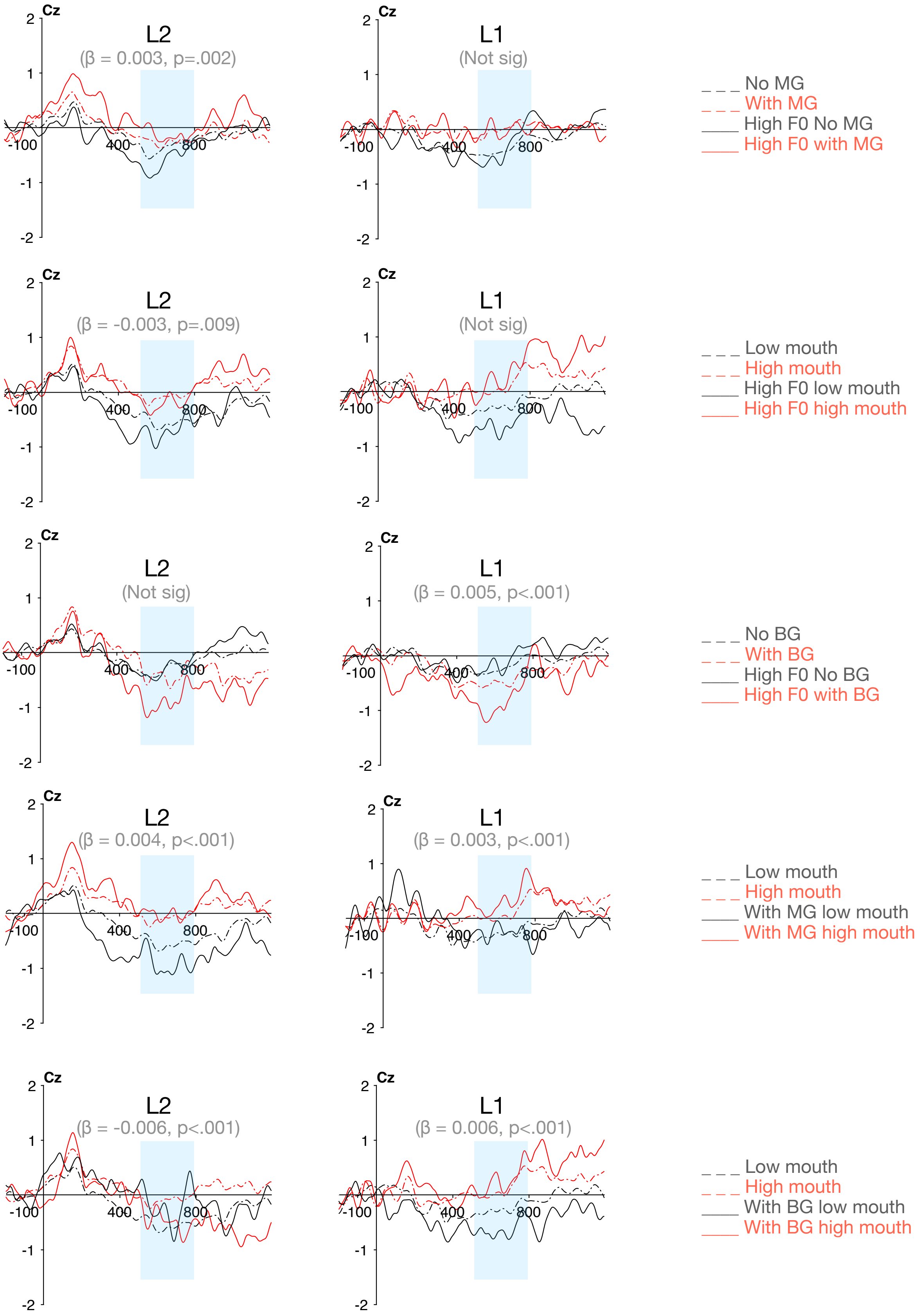

Figure S2. Interaction between surprisal and multimodal cues in L2 and L1. The effect of surprisal was modulated by pitch prosody, mouth movements and meaningful gestures in L2 users. Higher pitch prosody induced facilitatory effect (less negative N400) for L1 but not L2 group, while the facilitatory effect of informative mouth movements and meaningful gestures were larger for L2 groups.

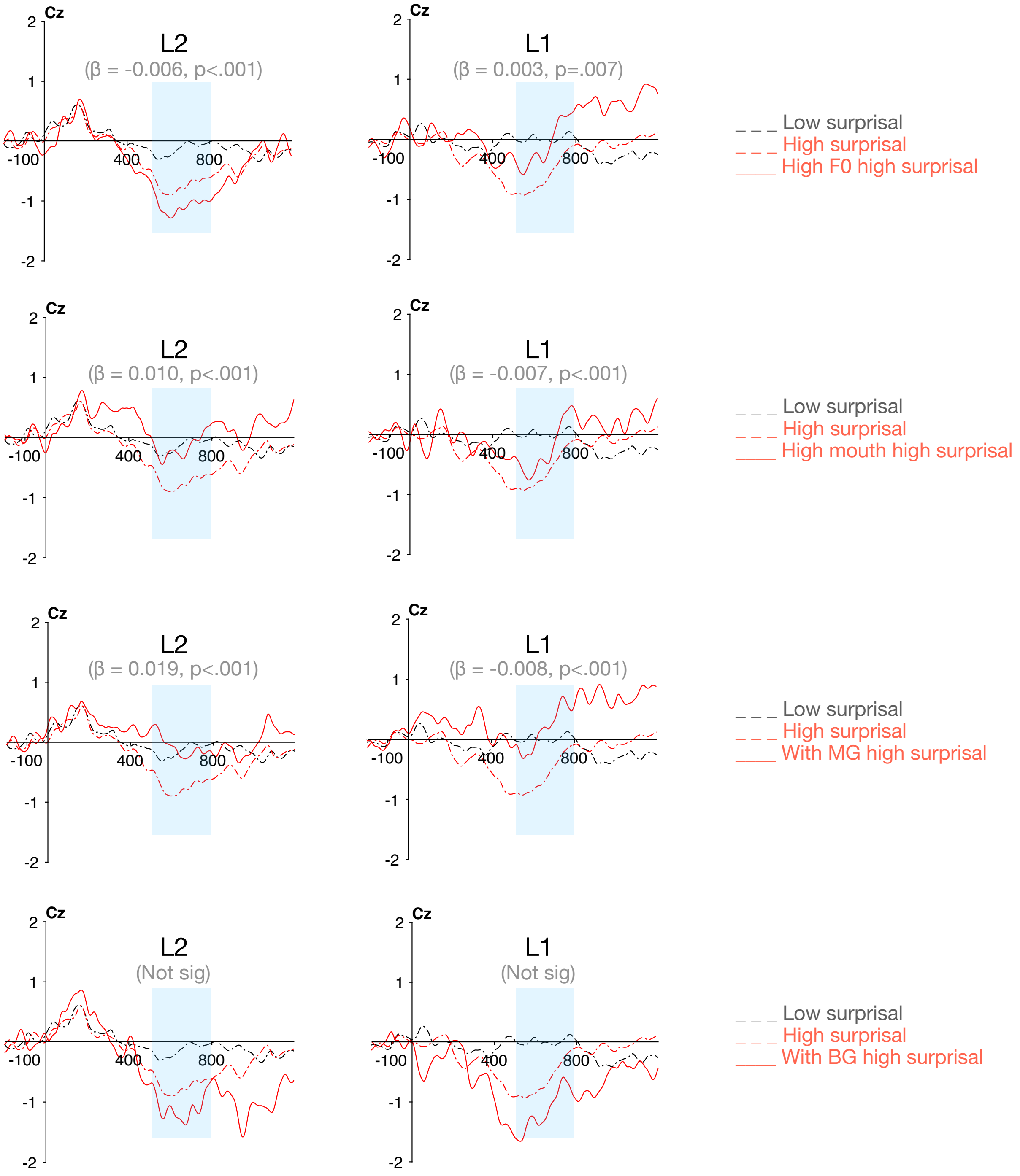

Figure S3. Interactions between multimodal cues in L2 and L1 groups. For L2 users, higher pitch prosody enlarged the facilitatory effect of meaningful gestures but not mouth; presence of meaningful gestures but not beat gestures enlarged the effect of mouth movements. L1 participants showed overall larger facilitatory effects of interaction between cues.

3. Selection of Time Window

Previous studies found that L2 participants exhibit a delayed N400 response compared to L1 comprehenders (e.g. Newman, Tremblay, Nichols, Neville & Ullman, 2012). Therefore, the current study conducted LIMO analysis for each group to identify the temporal distribution of N400. We found that for L2 participants, words with higher surprisal values showed more negative EEG signal in the approximately 500-800ms time window. Therefore, we used EEG signal in 500-800ms window as N400 for L2 in the main text. Conversely, the L1 group exhibited an N400 response in a slightly earlier time window, approximately 350-850ms, in line with previous findings (see panel A, Figure S4). We further combined L1 and L2 participants and carried out another LIMO analysis, which identified 400-850ms as shared N400 time window across both groups (see panel B, Figure S4)

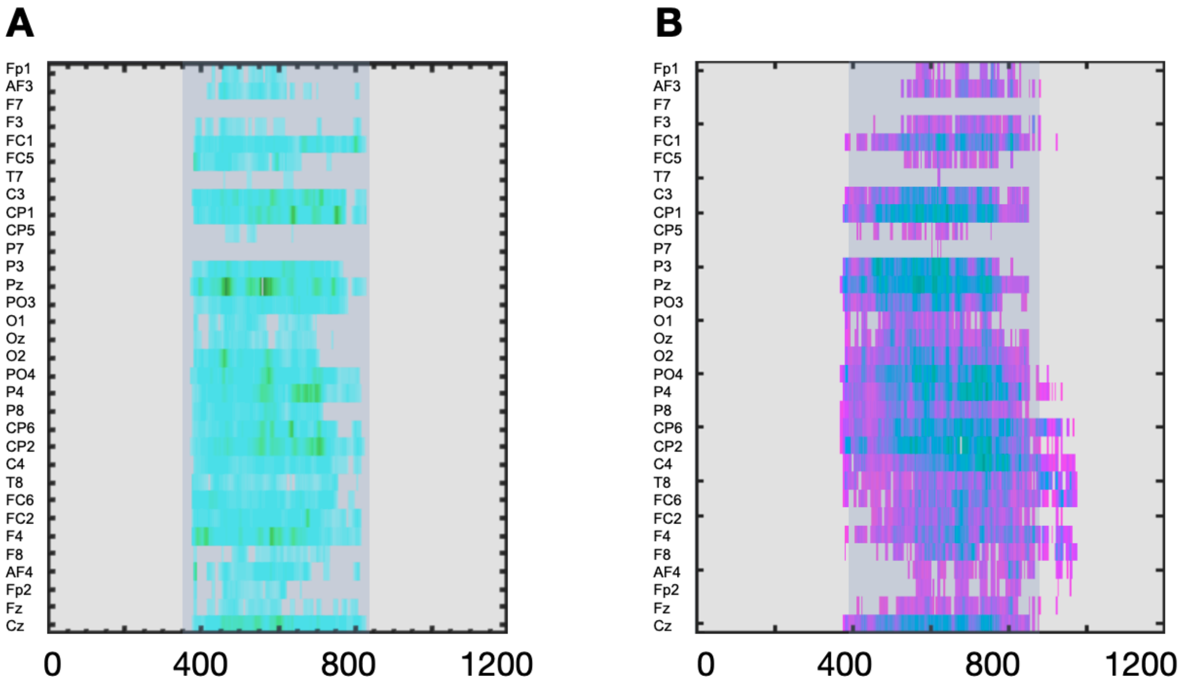

Figure S4. Time window sensitive to surprisal in L1 (panel A) and the combined sample of L1 and L2 (panel B).

In Analysis 2 in the main text, we carried out an LMER analysis in the 500-800ms time window for L1 and L2 participants (to be consistent with Analysis 1 in the main text). Additionally, we explored alternative time window comparisons to test the robustness of our result: 1) L1 participants in 350-850ms time window and L2 participants in 500-800ms time window (see Table S3); and 2) L1 and L2 participants in 400-850ms time window (see Table S4). The results are very similar with only numerical differences. The only exception is the interaction between native status, beat gestures and surprisal, being significant in the main text model but not in analysis 2) and 3). Therefore, we did not interpret this effect in the main text.

Table S3. *Full result: LMER model on N400 for L1 (350-850ms) and L2 (500-800ms) group.*

**Fixed Effects**	**β**	**Std Error**	**t**	**p**
(Intercept)	-0.002	0.007	-0.272	0.787
**Predictor Variables (effects of cues)**				
Native Status (Native)	-0.003	0.007	-0.433	0.667
Surprisal	-0.015	0.001	-13.407	<.001***
Mean F0	0.009	0.001	8.343	<.001***
Mouth Informativeness	0.011	0.001	11.059	<.001***
Meaningful Gesture (Present)	0.004	0.001	5.269	<.001***
Beat Gesture (Present)	-0.003	0.001	-3.439	0.001**
Surprisal:Mean F0	-0.001	0.001	-0.755	0.450
Surprisal:Mouth Informativeness	0.003	0.001	2.930	0.003**
Surprisal:Meaningful Gesture (Present)	0.012	0.001	13.325	<.001***
Surprisal:Beat Gesture (Present)	-0.002	0.001	-1.553	0.120
Mean F0:Mouth Informativeness	-0.002	0.001	-2.874	0.004**
Mean F0:Meaningful Gesture (Present)	0.004	0.001	5.390	<.001***
Mean F0:Beat Gesture (Present)	0.007	0.001	6.604	<.001***
Mouth Informativeness:Meaningful Gesture (Present)	0.007	0.001	8.921	<.001***
Mouth Informativeness:Beat Gesture (Present)	0.000	0.001	0.500	0.617
Surprisal:Mean F0:Mouth Informativeness	-0.003	0.004	-0.951	0.347
Surprisal:Mean F0:Meaningful Gesture (Present)	0.004	0.003	1.309	0.198
Surprisal:Mean F0:Beat Gesture (Present)	-0.001	0.004	-0.247	0.806
Surprisal:Mouth Informativeness:Meaningful Gesture (Present)	0.001	0.003	0.506	0.616
Surprisal:Mouth Informativeness:Beat Gesture (Present)	0.004	0.004	1.002	0.322
**Predictor Variables (interactions with native status)**				
Native Status (Native):Surprisal	-0.008	0.001	-7.649	<.001***
Native Status (Native):Mean F0	0.005	0.001	4.552	<.001***

Native Status (Native):Mouth Informativeness	0.004	0.001	4.380	<.001***
Native Status (Native):Meaningful Gesture (Present)	0.003	0.001	3.160	0.002**
Native Status (Native):Beat Gesture (Present)	-0.005	0.001	-5.756	<.001***
Native Status (Native):Surprisal:Mean F0	0.005	0.001	4.275	<.001***
Native Status (Native):Surprisal:Mouth Informativeness	-0.007	0.001	-6.494	<.001***
Native Status (Native):Surprisal:Meaningful Gesture (Present)	-0.007	0.001	-7.577	<.001***
Native Status (Native):Surprisal:Beat Gesture (Present)	-0.002	0.001	-2.280	0.023*
Native Status (Native):Mean F0:Mouth Informativeness	0.001	0.001	0.851	0.395
Native Status (Native):Mean F0:Meaningful Gesture (Present)	0.001	0.001	1.412	0.158
Native Status (Native):Mean F0:Beat Gesture (Present)	0.005	0.001	5.339	<.001***
Native Status (Native):Mouth Informativeness:Meaningful Gesture (Present)	0.003	0.001	3.965	<.001***
Native Status (Native):Mouth Informativeness:Beat Gesture (Present)	0.006	0.001	6.636	<.001***
Native Status (Native):Surprisal:Mean F0:Mouth Informativeness	-0.001	0.004	-0.266	0.791
Native Status (Native):Surprisal:Mean F0:Meaningful Gesture (Present)	-0.007	0.003	-2.269	0.029*
Native Status (Native):Surprisal:Mean F0:Beat Gesture (Present)	-0.001	0.004	-0.137	0.891
Native Status (Native):Surprisal:Mouth Informativeness:Meaningful Gesture (Present)	-0.006	0.003	-2.169	0.036*
Native Status (Native):Surprisal:Mouth Informativeness:Beat Gesture (Present)	0.001	0.004	0.258	0.798
**Control Variables**				

Word Order	0.002	0.001	3.327	0.001**
Word Length	0.005	0.001	5.997	<.001***
Sentence Order	-0.001	0.001	-1.075	0.282
Baseline	0.763	0.001	1100.835	<.001***
Electrode X	-0.006	0.001	-9.124	<.001***
Electrode Y	0.003	0.001	4.970	<.001***
Electrode Z	-0.004	0.001	-6.509	<.001***

**Random Effects**		**Variance**	**Std.Dev.**
Participant ID	(Intercept)	0.002	0.041
	Surprisal:Mean F0:Mouth Informativeness	0.000	0.022
	Surprisal:Mean F0:Meaningful Gesture (Present)	0.000	0.020
	Surprisal:Mean F0:Beat Gesture (Present)	0.001	0.024
	Surprisal:Mouth Informativeness:Meaningful Gesture (Present)	0.000	0.015
	Surprisal:Mouth Informativeness:Beat Gesture (Present)	0.000	0.022

**Model**				
AIC	BIC	logLik	deviance	df.resid
1738353	1739160	-869108	1738215	883515

Notes. * $p < .05$, ** $p < .01$, *** $p < .001$

Table S4. *Full result: LMER model on N400 for L1 and L2 group in 400-850ms.*

**Fixed Effects**	**β**	**Std Error**	**t**	**p**
(Intercept)	-0.001	0.006	-0.241	0.810
**Predictor Variables (effects of cues)**				
Native Status (Native)	-0.005	0.006	-0.809	0.423
Surprisal	-0.015	0.001	12.914	<.001***
Mean F0	0.009	0.001	8.682	<.001***
Mouth Informativeness	0.012	0.001	12.187	<.001***
Meaningful Gesture (Present)	0.004	0.001	5.220	<.001***
Beat Gesture (Present)	-0.002	0.001	-2.654	0.008**

Surprisal:Mean F0	-0.001	0.001	-1.162	0.245
Surprisal:Mouth Informativeness	0.004	0.001	3.419	0.001**
Surprisal:Meaningful Gesture (Present)	0.011	0.001	12.331	<.001***
Surprisal:Beat Gesture (Present)	-0.001	0.001	-1.448	0.148
Mean F0:Mouth Informativeness	-0.002	0.001	-2.146	0.032*
Mean F0:Meaningful Gesture (Present)	0.004	0.001	5.592	<.001***
Mean F0:Beat Gesture (Present)	0.006	0.001	6.321	<.001***
Mouth Informativeness:Meaningful Gesture (Present)	0.008	0.001	9.427	<.001***
Mouth Informativeness:Beat Gesture (Present)	0.001	0.001	1.426	0.154
Surprisal:Mean F0:Mouth Informativeness	-0.003	0.004	-0.947	0.350
Surprisal:Mean F0:Meaningful Gesture (Present)	0.003	0.003	0.974	0.336
Surprisal:Mean F0:Beat Gesture (Present)	-0.002	0.004	-0.539	0.593
Surprisal:Mouth Informativeness:Meaningful Gesture (Present)	0.001	0.003	0.506	0.616
Surprisal:Mouth Informativeness:Beat Gesture (Present)	0.005	0.004	1.362	0.180
**Predictor Variables (interactions with native status)**				
Native Status (Native):Surprisal	-0.010	0.001	-9.615	<.001***
Native Status (Native):Mean F0	0.005	0.001	4.628	<.001***
Native Status (Native):Mouth Informativeness	0.004	0.001	3.992	<.001***
Native Status (Native):Meaningful Gesture (Present)	0.003	0.001	3.146	0.002**
Native Status (Native):Beat Gesture (Present)	-0.007	0.001	-7.377	<.001***
Native Status (Native):Surprisal:Mean F0	0.004	0.001	3.198	0.001**
Native Status (Native):Surprisal:Mouth Informativeness	-0.008	0.001	-7.131	<.001***
Native Status (Native):Surprisal:Meaningful Gesture (Present)	-0.006	0.001	-6.683	<.001***

Native Status (Native):Surprisal:Beat Gesture (Present)	-0.002	0.001	-2.426	0.015*
Native Status (Native):Mean F0:Mouth Informativeness	0.000	0.001	0.110	0.912
Native Status (Native):Mean F0:Meaningful Gesture (Present)	0.001	0.001	0.776	0.438
Native Status (Native):Mean F0:Beat Gesture (Present)	0.006	0.001	5.693	<.001***
Native Status (Native):Mouth Informativeness:Meaningful Gesture (Present)	0.003	0.001	4.120	<.001***
Native Status (Native):Mouth Informativeness:Beat Gesture (Present)	0.005	0.001	6.205	<.001***
Native Status (Native):Surprisal:Mean F0:Mouth Informativeness	-0.002	0.004	-0.433	0.667
Native Status (Native):Surprisal:Mean F0:Meaningful Gesture (Present)	-0.007	0.003	-2.279	0.028
Native Status (Native):Surprisal:Mean F0:Beat Gesture (Present)	-0.000	0.004	-0.126	0.900
Native Status (Native):Surprisal:Mouth Informativeness:Meaningful Gesture (Present)	-0.005	0.003	-2.157	0.037*
Native Status (Native):Surprisal:Mouth Informativeness:Beat Gesture (Present)	0.000	0.004	0.037	0.971
**Control Variables**				
Word Order	0.002	0.001	3.519	<.001***
Word Length	0.005	0.001	6.245	<.001***
Sentence Order	-0.000	0.001	-0.391	0.696
Baseline	0.766	0.001	11.678	<.001***
Electrode X	-0.006	0.001	-9.128	<.001***
Electrode Y	0.004	0.001	5.154	<.001***
Electrode Z	-0.004	0.001	-6.148	<.001***

**Random Effects**		**Variance**	**Std.Dev.**
Participant ID	(Intercept)	0.001	0.039

Surprisal:Mean F0:Mouth Informativeness	0.000	0.022
Surprisal:Mean F0:Meaningful Gesture (Present)	0.000	0.020
Surprisal:Mean F0:Beat Gesture (Present)	0.001	0.023
Surprisal:Mouth Informativeness:Meaningful Gesture (Present)	0.000	0.015
Surprisal:Mouth Informativeness:Beat Gesture (Present)	0.000	0.021

**Model**				
AIC	BIC	logLik	deviance	df.resid
1728179	1728986	-864021	1728041	883515

Notes. * $p < .05$, ** $p < .01$, *** $p < .001$

4. Full stimuli

1	But one person here knew him differently to the rest of us. And the family would like to invite him now to read a short passage, chosen from the book Dr Rafferty was reading when he died.
2	The wedding ring, ten years old at least. The rest of her jewellery has been regularly cleaned, but not her wedding rings - state of her marriage, right there. The inside of the rings are shinier than the outside - that means they're regularly removed; the only polishing they get is when she works them off her finger. It's not for work - look at her nails, she doesn't work with her hands - so what, or rather who, does she remove her rings for? Clearly not one lover - she'd never sustain the fiction of being single over time - so more likely a string of them.
3	Why do they make that noise? It's only a tennis ball it's not very heavy. It's not like they're hitting a rock or a small something made from osmium, which is the heaviest metal known to man.
4	What? I assure you, Hannay, tomorrow it will be impossible either to steal or copy those plans. The First Sea Lord, Prince Louis of Battenburg, will present them to the committee, then they'll be locked away under guard.
5	So the question I'd like to ask is this - when a drug crazed maniac breaks into your house with a knife, or God forbid, two knives, what do you do? WHAT DO YOU DO?
6	Well why don't you go and move into the middle of a field then? Build a great big wall all around yourself, so you won't have to be contaminated by any of us lot from the Clampmead!
7	Working out if someone's lying isn't an exact science. But there are things liars do, like unconsciously touch their face, their mouth. The only time I felt Arkady said something untrue was when I asked why he was turning.
8	You dragged him out and you beat him. You didn't hit him in the head or the face - you didn't want anyone to see the marks as you dragged him out of the building. No - you hit him in the back...
9	Ben, you were specifically told 'no fighting, no swearing and no drinking alcohol' and I caught you bashing that kid's head on the floor and shouting 'Don't touch my bloody beer'.
10	See that drive up to the timeshare, the scenery is absolutely beautiful, see the hills, the lochs, the windfarms, just beautiful. That last time we went up, the weather was smashing so we drove the whole way with the roof down. It was gorgeous so it was.
11	You didn't know that Simone would routinely try to beat the Devil out of her with a belt while Ida stood by and watched? Maybe you can tell me which part of the Bible sanctions that?
12	I've been looking for hours. Nothing. You know if he's been knocked over someone could have lasoed him to the back of a car, driven through the tunnel and dumped in a bin somewhere. Or thrown him off the Pier Head.
13	Your husband will be home soon. Either he opens the door and finds his wife and baby spread all over the carpet - or he comes home to find the baby asleep in her cot and his wife gone.
14	It sounded better in my head. It was on David Attenborough. One bird just sits there, proud as you like, while the other digs at it with its beak, removing fleas and microscopic ticks. So it's very loving. But also, the clever thing, dinner, sorted.
15	And he shows me a Polaroid of Grace Allen. And she's alive. Nearly two years later, she's still alive. In the picture, right next to her face, he's folded up that day's paper. Just to prove it. He says, "this is the closest you'll ever come." And then he sets light to the picture. Burns it to ashes, right in front of me. Then he cut me loose.
16	And the students will learn to part those clouds, to throw their gaze beyond mortal things, to reach the eternal part of themselves and finally see the face of God.
17	All right, let me get this straight, DS Arnott – what you're saying is that some impostor acquired the firearm and then tampered with the paperwork to cover his tracks?
18	The decision appeared to make sense at the time. There were roadworks on Crown Avenue, with temporary traffic signals controlling a single lane. It was a potential hold-up that would've left us vulnerable.
19 (practice)	Andy we can't turn up to a property auction with ten grand in our pocket. That buys a deposit on a flat, not a three bedroom cottage in half an acre of land.
20	When was the last time you did anything truly practical, Mrs. Grant? Like milked a cow. Or slaughtered a pig. Or grew a vegetable. Do you know which mushrooms are okay to eat and which ones will kill you? That's the kind of knowledge you're going to need now.
21 (practice)	I didn't want to put pressure on you getting a job. I was going to tell you as soon as you got work but the weeks and months are passing and you're still signing on.
22 (practice)	But Steve is so determined. I remember once we were at Center Parcs and a horse trod on my foot and wouldn't get off. Steve kept punching it in the face until it moved. You don't forget that kind of loyalty in a man.
23	I'm advising you Jonathan. You fly that girl out, it's a confession of her guilt. Freddie Hamid will know it was her. So will Richard Roper. And no one will lift a finger to stop them.

24 (practice)	The most recent records are consistent with your testimony, DCI Gates. Calls and texts from Ms. Laverty directed to your phone. None of the calls answered by you. One-way traffic. Go back a little further, and you're calling her. Texting her. Would you like me to read an example of one of the text messages sent by you to Ms Laverty last month?
25	Materials that survived the explosion suggest that the device was housed in an airtight container. When the detonator was triggered, it could've opened a valve to allow in oxygen to support combustion of the TATP explosive.
26	When I was in France, there were these men, I'd loosely call them journalists. Never went to the front line, never really put their head above the parapet, terrified of getting shot. Yet they wrote some of the best battle reportage I've ever read, filching from the rest of us.
27	It was north of Kirknairn, approximately half a mile after the left hand turning with the gravel heap on the side of the road and the beech tree on the right.
28	We'd literally just moved in, we were still living out of boxes. It was dark, I hadn't sorted out the wiring yet. She was at the top of the stairs and... They said she must have fallen awkwardly.
29	I just keep taking the folic acid and iron tablets... It's the worst thing living in a grotty student pad when you're used to living in a five bedroom detached house with a cleaner and a dishwasher.
30	I understand. It's just that the photo - it's our dad, Arthur. He died only four months later. The moment he heard about Eric he just broke. Wouldn't eat, drink - nothing. Mum never got over it.
31	Forget this place. Let's go to Pound Kingdom. You could buy Judy thirty different presents. Hair clips, cheap foreign chocolate, a washing frame, 100 disposable hair nets, nougat, marigolds, engine lubricant, a wrench set, an out of date calendar, baby food, batteries, pasteurised meats...
32	The barbecue was still hot, so, we advised them to leave the barbecue at the side of the road, wait until it cooled down, and then come back and pick it up a little bit later on.
33	You served half your sentence and now you're out on licence. You understand all this, of course. We want to help you move on but it's not gonna be easy. You're gonna need money for rent, for living expenses.
34	I'll drive you! That's what I'll do. Can't have you traipsing around town with a heavy suitcase, it's not right. Are you still in that Islington flat? I'll pick you up, 8 o'clock sharp tomorrow morning, there's a good girl.
35	Sarah gave me her key yesterday, asked me to pick something up. And before you ask - no, I don't know where she is - but I'm pretty sure she wouldn't like you in her room.
36	We were having a night in. Just watching a DVD. He usually falls asleep, you know but he sat through this one. He was...quiet. Out of the blue he said he had to go and see someone.
37	I'm a kinder-transport child. Great Britain took me in - I was thirteen years old. My family stayed behind in Germany. They were murdered in the camps - every one of them. I owe my new country everything including my life so once a year when everyone else is busy pulling crackers and eating turkey I take the time to contemplate my good fortune, the debt I owe and the importance of doing small things well.
38	But how did Maksim's body get from here to the car? There are security guards on every level but the basement. There's no sign anything was dragged to the lift, so unless every guard in this building was in on the plan, how did they do it?
39	According to the prison officers, their transporter was forced off the road by the vehicle you see there, driven by two men in motorcycle gear, same as the ambush. Denton made her escape.
40	Once the crush had developed at the turnstiles the police opened an exit gate to relieve the pressure. Two thousand fans went through this gate and followed the sign in front of them to the already crowded pens on the terrace.
41	I had a friend once who had a cactus. For fifteen years he had that cactus sitting on his windowsill until one day someone bought him a second cactus. Within six months he had to move to a bigger house so that he had space for his cactus collection.
42	After all, it's just the way God made you. That's what your Gran always used to say - her weight never bothered her. She used to say to me, yes Peg, I'm nineteen stone, I can barely catch enough breath to butter a scone and it takes two of the neighbours to winch me on and off the toilet but I wouldn't have it any other way.
43	He tried to kill you, Catherine. You're not over reacting. We'll send it all off for finger prints, and I'll ring the prison liaison officer to talk to someone at Gravesend. Let's find out who visits him, who he writes to, who he has phone calls with. If he is behind it, he'll be dealt with. And if it is a crank, it's a shame they've got nothing better to do.
44	She'd only gone and written her number on the back of the lottery ticket. I mean if the lad had just opened the bit of paper up and seen what it was, he'd have been worth a fortune.
45	I went to Tim's flat to challenge him, that's all. I covered my tracks purely in case he decided to raise an official complaint. Things became heated. There was a struggle. I hit my head. Blacked out. And when I came round, he was in his forensic over suit. I was laid out on plastic sheeting, and he had tools to dismember my body...

46	Tiny amounts of carpet fibres are thrown into the air and settle on objects in the immediate vicinity. This gives an indication of how long the objects have been in said location.
47	She told her to put all her troubles in little envelopes. And to put all the little envelopes in a little box. And to put it in the attic until she felt strong enough to open it up again.
48	I've made a dozen bottles of nettle wine for our celebration and I was wondering if you'd all like to come round to the gatehouse after work so we could all watch the draw together.
49	We dropped my car at his mate's garage, and then he gave me a lift home. The next morning, he called round so we could drop Nathan at nursery. But instead of going to collect my car, Joe said we were going to the hospital. He said he knew people who'd kill Nathan unless I did what they needed me to do.
50	This is really serious, Catherine. You have got a case to answer! You found the body, you made threatening phone calls, you had a motive, and now you've turned up at the victim's funeral!
51	Part of being a mum is being able to just handle whatever gets thrown at you. I've got this mug I bought myself one Mother's Day that says "Keep Calm and be a Mum". That's what I live by. Whatever happens, I will always look after Angel - make sure she's going to school, not getting fat or anything... And if one day I look down and I notice that she's getting a bit chunky or whatever I'll always know what to do. Keep calm and be a mum.
52	Okay well then I'm going to have to ask you to give me your keys to your vehicle. You'll understand that I can't let you drive away from the scene if you're refusing to be breathalysed.
53	Tommy, I know you hate parties and people and talking about nothing, but it's my wedding day so you'll bloody well grin and bear it and stop looking at your watch. You understand?
54	Remember my first fight? I was thirteen. Hammered the guy. Looked over, fists in the air, but you weren't watching. You were cutting a deal. Didn't even notice I'd won. You never did give me a chance.
55	The staging area for the police was just here. We were called up for safety - no one expected any trouble. As the morning wore on we had to bring more shifts in. Had reports there were people occupying a building further along.
56	"Possibly." Look. We all know under modern policing methods it's extremely difficult. It's nigh on impossible to plant evidence once a crime scene has been opened, however it is conceivably possible to replace evidence once it's in storage if one has access to said storage facility.
57	We had a few cross words. She said some very naive things about new build properties and then walked out. Now, it's raining, her phone's off - I don't know where she's gone.
58	Oh the milk tray man was the perfect man. Strong, mysterious, swam through shark infested waters to get to the woman he loved. Oh I used to dream about him. He'd shimmy up my drainpipe, knock on my window and I'd be there waiting with my eyes closed, my mouth open.
59	The caller insisted they page the nurse but they wouldn't because she wouldn't give a name. She hung up. By the time they reported it, the Witness was already dead.
60	I've heard back from the traffic boys, this morning. They think Evan must have slipped out of town on one of the back roads - they can't pick him up on any cameras.
61	Marijuana. So under the influence of drugs you turned up - unannounced - to see your children. You upset the children. And then you fled to a pub, drank to oblivion and fell in front of a fast moving vehicle.
62	If I let go of your neck, you'll die. Which is what you want, right?... But if I keep applying pressure like this, there's a pretty good chance you'll live.
63	Chrissie, can I just say... I hope that at some point in the future we can just sit down, open a big bottle of wine, stick on a romcom and have a a proper girly chat, like a couple of mates. What do you think?
64	Listen to me - I'm going to move you. I'm going to get you to the hospital. You bloody well stay with me, Ed. You hear? Ready? Here we go.
65	Okay. Can I just say this though, Liam. The lighter's making me nervous. You've had a lot to drink and you've got the shakes and you might press it without intending to, and I'd like you to put it down.
66	If this was done by an amateur, the head would have been pulled back, like you see in the movies. Whoever did this has military training or experience of hand to hand combat.
67	Wolfbloods are invisible. We walk in the margins of the world. We move without sound, we follow scents but leave none of our own. We are shadow and night and the bright strength of the moon. We are beyond their understanding.
68	Look at that. All this space... and we choose to live crammed into a few square miles. High-rise buildings, gardens barely big enough for a barbeque and a few chairs, knees knocking against each other under the table...
69	I'm gonna write you a cheque for ten thousand pounds. You can do whatever you like with it. Go travelling. Sit on your arse. Spend it on booze or birds or swimming with dolphins. Use it to figure out what you're going to do with your life. But stop wasting everyone's time trying to be like your sister. You've got so much more potential than she ever had. Don't throw it away.

70	And the upstairs loo needs a good scouring as well. I always check under the seat, just to see how clean it is. Well I lifted the seat and what did I see?
71	That’s something you’re going to have to learn. You take the pressure in the daytime, but when the heads hits the pillow, oblivion. Or you’ll never last. Not in this game.
72	He was face down when they found him. My guess is he was pulling himself in this direction when he had his throat cut. You can see from the drag patterns, the blood saturation...
73	Listen darling, I’m just going to pop down to the station to pick up a chum who’s running late and I can’t seem to find my keys. You haven’t had them have you?
74	Nah, I’m good. But if you’re still sitting out here when I clock off, you can give me a ride back to Dadir’s. That’s where I’m sleeping now that I’m technically homeless. Thanks for that by the way.
75	He'll find a way. When he calls, give him a message. There's a subway between Borogrove Estate and Moss Heath Park. You tell him I'll be there, alone, at midnight.
76	Zebedee ran past Dad and nearly knocked him over. “I’m sure that dog’s getting bigger by the minute”, said Dad. “He’s going to eat us out of house and home”. Ben and Albert laughed. If only Dad knew.
77	Rule Number One - don’t get yourself in this situation again. Which means, you observe case management protocols. Any proactive strategies are to be signed off by me. I don’t sign, they don’t happen.
78	Ron was one of the good guys. But, I think it broke him, in the end... that it happened on his watch. Ended up shutting the place down anyway, while Gower swanned off back to his estate.
79	I know, I’m just a greedy bastard. I’ve cashed up till two. I’ll just mark down the sell-by’s and then I’ll get off. Will you be alright to cash up till one and lock up?
80	Yeah, he’s basically saying I was a maverick. Sometimes you have to throw away the rule book and go with a hunch. I wasn’t in the business of bureaucracy and red tape, I was in the business of catching criminals.
81	The line of the cut is jagged, meaning the skin and muscle was slack when it was cut. If you pull someone’s head back to cut their throat, the skin would be taut and the cut straight. So...
82	It smells wonderful. You know, you can move in, decorate and furnish a place all you like. But a home is not a home until it is filled with the aromas of food on a stove.
83	The eggs don’t actually go anywhere. The sperm move towards the eggs. It’s not like a game of “It” with sperm and eggs chasing each other round the fallopian tubes.
